# Exploration of immune-related cells and ceRNA in squamous cell lung cancer

**DOI:** 10.1097/MD.0000000000027058

**Published:** 2021-09-03

**Authors:** Lijun Wang, Hao Wang, Ke Xu, Yehong Xu, Yong Wang, Song Wei, Zhihong Zhang

**Affiliations:** a Department of Respiratory Disease, Building 8 of Tongling People's Hospital, Tongling; b Department of Respiratory Oncology, Anhui Provincial Cancer Hospital (The First Affiliated Hospital of USTC West District), Hefei; c Department of Respiratory Disease, The Fifth People's Hospital of Fuyang City, Fuyang, Anhui, P.R. China.

**Keywords:** ceRNA, CIBERSORT, prognosis, SqCLC, TCGA

## Abstract

The treatment for squamous cell lung cancer (SqCLC) is limited, and the prognosis of SqCLC is poor. In this article, we aimed to analyze and identify immune-related cells and competition endogenous RNA (ceRNA) that influence the prognosis of SqCLC. SqCLC and lung adenocarcinoma data were downloaded from TCGA-GDC. A total of 22 types of immune cell fractions were estimated using CIBERSORT. R software was used to identify any significantly different transcriptome data, including mRNA, LncRNA, and miRNA. The univariate cox regression method was applied to screen for prognosis-related lncRNA, miRNA, mRNA and tumor-infiltrating immune cells. There were 504 patients included in this study. There was a higher proportion of memory activated CD4+ T cells and CD8+ T cells in younger women. Follicular helper T (Tfh) cells were predictive of a good prognosis and reflected immune activation in SqCLC. The SFTA1P/NKX2-1-AS1, hsa-mir-503, GREM2 ceRNA axes and NKX2-1-AS1, hsa-mir-96, PROK2 ceRNA axes were found to be important for the immune function, pathogenesis, and prognosis of SqCLC. Collectively, the immune-related ceRNA and tumor-infiltrating immune cells in SqCLC are likely important determinants of SqCLC pathogenesis, prognosis, and immune status.

## 1 Introduction

Lung cancer is the leading cause of cancer-related death worldwide and approximately 85% of lung cancers are non-small cell lung cancer (NSCLC).^[1]^ Squamous cell lung cancer (SqCLC) represents around 20% to 30% of all NSCLC. In addition, SqCLC has specific clinicopathologic characteristics, including the central location of tumors, diagnosis at an advanced stage, older age, and presence of comorbidities (eg, chronic obstructive pulmonary disease, cardiovascular disease, and diabetes), which present limitations and challenges to treatment. Due to a lack of genetic alterations with approved targeted treatments, chemotherapy is the primary first-line treatment for SqCLC. The discovery of immune checkpoint inhibitors, particularly inhibitors of the tumor programmed death 1 (PD-1) axis, provide critical options for patients with SqCLC in whom PD-L1 expression ≥50%.^[2]^ However, the prognosis of SqCLC remains extremely poor.

Many kinds of immune cells and immune-related genes are related to immune checkpoint inhibitor, such as, tumor mutation burden, the presence of tumor infiltrating lymphocytes (TILs).^[3]^ Recent studies indicate that high tumor-infiltrating CD 68-positive cells are correlated with better progression-free survival for lung adenocarcinoma.^[4]^ Based on TCGA data set, Kuo Hao Ho and so on have found that high proportion of tumor infiltrating B cells is associated with better prognosis of NSCLC.^[5]^ Moreover, immune gene participate in brain metastasis in NSCLC.^[6]^ In addition, patients with high expression of immune gene PSMB9 showed longer PFS in NSCLC.^[7]^ However, since the immune-related cells and competition endogenous RNA (ceRNA) in SqCLC not fully research, there is a need to understand the factors associated with the immune mechanism, pathogenesis, and prognosis of SqCLC.

In this study, we report that there was a higher proportion of CD8+ T cells and memory-activated CD4+ T cells in younger women with SqCLC. We further showed that follicular helper T (Tfh) cells were predictive of a good prognosis and reflected immune activation in SqCLC. Moreover, the SFTA1P/NKX2-1-AS1, hsa-mir-503, GREM2 ceRNA axes and NKX2-1-AS1, hsa-mir-96, PROK2 ceRNA axes were found to be important for the immune function, pathogenesis, and prognosis of SqCLC.

## 2 Materials and methods

### 2.1 Data processing and differential expression analysis

Genomic and clinical data were downloaded from The Cancer Genome Atlas (TCGA; https://portal.gdc.cancer.gov/). The RNA expression data included 502 tumor samples and 49 normal samples, the miRNA expression data contained 478 neoplasm samples and 45 normal samples. A total of 504 clinical data were obtained. We used the HUGO gene Nomenclature Committee (HGNC; http://www.genenames.org/) to annotate the lncRNA and mRNA genes for RNA expression profiling. The R (version 3.5.3; https://cran.r-project.org/) software limma package (version 3.47.2; http://www.bioconductor.org/packages/devel/bioc/html/limma.html) was used to normalize and analyze the data we collected. Finally, we obtained the data regarding the differentially expressed mRNA, lncRNA, and miRNA molecules.

### 2.2 Estimation of the immune cell type fraction

CIBERSORT (https://cibersort.stanford.edu/) is a versatile computational method for quantifying the cell fractions from bulk tissue gene expression from the purified leukocyte subset, and has been confirmed by flow cytometry.^[8]^ The LM22 gene signature contains 547 genes which can discriminate between the 22 human hematopoietic cell phenotypes (B cells, T cells, natural killer cells, macrophages, dendritic cells, and myeloid subsets). The mRNA expression data were extracted from the RNA expression profile, which was downloaded from the TCGA datasets. The R software Limma package was used to normalize the mRNA data. The CIBERSORT, LM22 gene signature, and normalized mRNA were used to quantify the proportion of immune cells in the SqCLC. Patients with a CIBERSORT *P* < 0.05 were considered eligible for further analysis.

### 2.3 Association between TILs, clinical-pathological variables and prognosis

Age, grade, sex, Primary tumor (T), Regional lymph node (N), distance metastasis (M), stage, and overall survival (OS), were collected from the clinical data downloaded from the TCGA database. Kruskal-Wallis tests and Wilcoxon tests were used to test the relationship between TILs and clinical-pathological variables. The R software survival package and univariate cox regression method were used to screen for prognosis-related TILs.

### 2.4 Immune-related lncRNA-miRNA-mRNA regulatory network construction

A list of the genes involved in immune modulation was downloaded from the IMMPORT database(https://www.immport.org/shared/home). The intersection of the IMMPORT genes and differentially expressed genes (DEGs) was used as the immune-related DEGs (iDEGs). We applied pearson correlation analysis in the R software to identify the potential DElncRNA related with iDEGs as immune-related DElncRNA (iDElncRNA). If the correlation coefficient (|R|) >0.4 was considered as a strong correlation, and *P* < 0.01 was statistically significant. Based on the comprehensive GENCODE gene annotation, miRcode (http://www.mircode.org/) provides “whole transcriptome” human miRNA target predictions. The miRcode database was conducted to predict the interaction between iDElncRNAs and miRNAs. iDElncRNAs correlated with DEmiRNA were perfectly matched. The target DEmiRNA mRNAs were predicted using TargetScan (http://www.targetscan.org/), miRDB (http://www.mirdb.org/miRDB/), and miRTarBase (http://mirtarbase.mbc.nctu.edu.tw) online analysis tools. Furthermore, we used a Venny diagram to obtain the intersecting part that was used as the DEmRNA between the target mRNAs and iDEGs. We eventually obtained the relationship pair: DElncRNA-DEmiRNA and DEmiRNA-DEmRNA. We used cytoscape software (version 3.6.0; https://cytoscape.org/) to construct the lncRNA-miRNA-mRNA ceRNA network.

### 2.5 Prognosis values of DElncRNA, DEmiRNA, and DEmRNA in the ceRNA network

The survival package (version 1.2; https://cran.r-project. org/package=survival) and univariate cox regression method were used to screen the prognosis-related DElncRNA, DEmiRNA, and DEmRNA in the ceRNA network. We divide the expression of SFTA1P and NKX2-1-AS1 in tumor patients into high-expression group and low-expression group based on the average value. *P* < 0.05 was considered to be statistical significantly.

### 2.6 Relationship between ceRNA and TILs

Tumor Immune Estimation Resource (TIMER, https://cistrome.shinyapps.io/timer/), is a comprehensive resource for the clinical relevance of tumor-immune infiltrations. We uploaded GREM2 to explore the relationship among ceRNA and TILs.

### 2.7 Statistical analysis

The differential gene expression between SqCLC and the normal tissues was assessed using an unpaired *t* test. The association between tumor-infiltrating immune cells or immune related genes and the corresponding clinical follow-up were analyzed using Kaplan–Meier curves. *P* values of <.05 were considered significant.

## 3 Results

### 3.1 Identification of different level of the 22 types of TILs

**Figure 1:**
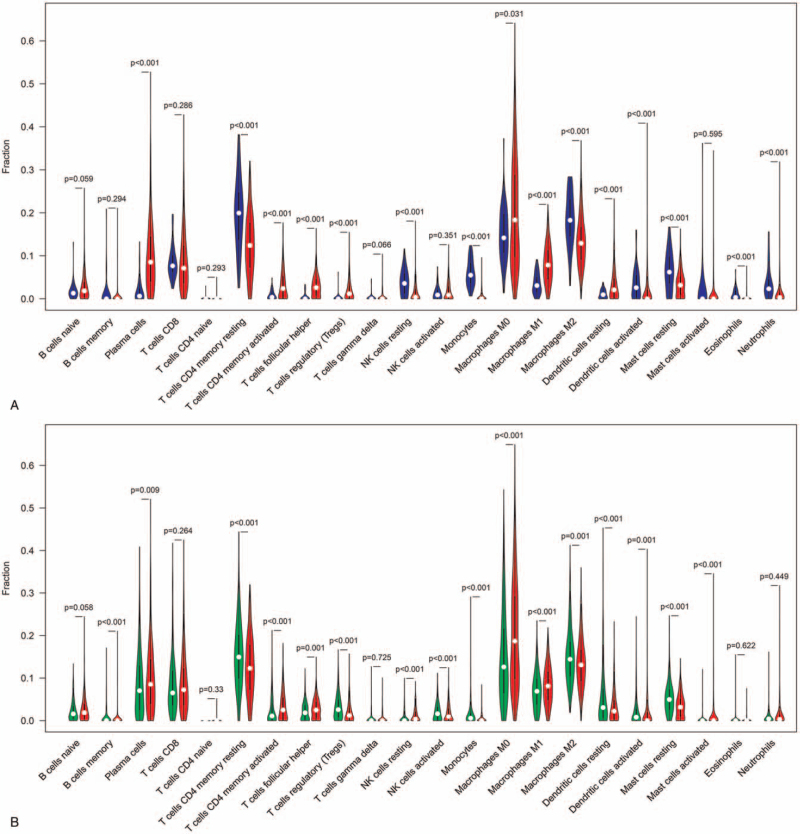
Violet plot of the 22 types of TILs in SqCLC. The percentage of plasma cells, CD4+ memory activated T cells, follicular helper T cells, M0 macrophages and M1 macrophages were significantly higher in SqCLC than both in the normal lung tissues and LUAD. The number of resting CD4+ memory T cells, NK cells, monocytes, M2 macrophages and mast cells were significantly lower in SqCLC than both in the normal lung tissues and LUAD. Red violet: SqCLC; Blue violet: normal lung tissues; Green violet: lung adenocarcinoma, LUAD. (A) The proportion of TILs in SqCLC versus normal lung tissues. (B) the proportion of TILs in SqCLC versus LUAD. LUAD = lung adenocarcinoma, SqCLC = squamous cell lung cancer, TILSs = tumor infiltrating lymphocytes

**Figure 2:**
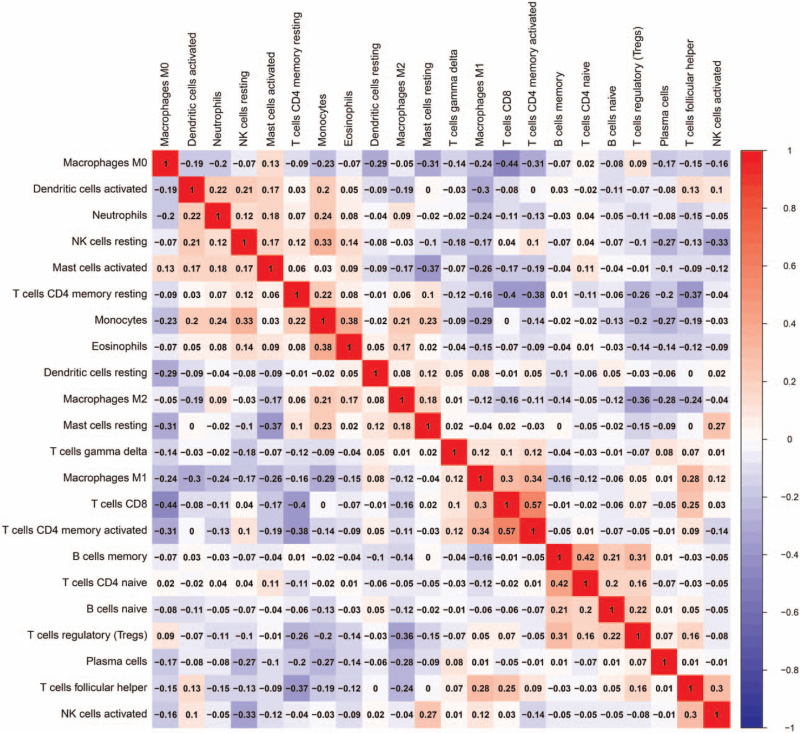
Correlation matrix of all 22 immune cell proportions. Tfh cells were positively correlated with NK cells activated (*r* = 0.299), CD8+ T cells (*r* = 0.25), CD4+ memory activated T cells (*r* = 0.09), regulatory T cells (*r* = 0.16), and M1 macrophages (*r* = 0.28).

To elucidate the differential level of the 22 TILs between SqCLC, lung adenocarcinoma (LUAD) and normal lung tissues, we constructed a violin plot, as shown in Figure 1. The violet plot showed the percentage of the 22 TILs as well as any statistical differences. The percentage of plasma cells, CD4+ memory activated T cells, follicular helper T cells, M0 macrophages, and M1 macrophages were significantly higher in SqCLC than both in the normal lung tissues and LUAD. The number of resting CD4+ memory T cells, NK cells, monocytes, M2 macrophages, and mast cells were significantly lower in SqCLC than both in the normal lung tissues and LUAD. This significant difference in TILs may provide novel research directions regarding the mechanism associated with the occurrence and development of SqCLC. Interestingly, we also found that Tfh cells was positively correlated with activated NK cells (*r* = 0.299; *P* < .01), CD8+ T cells (*r* = 0.25; *P* < .01), memory activated CD4+ T cells (*r* = 0.09; *P* = .048), regulatory T cells (*r* = 0.16; *P* < .01), and M1 macrophages (*r* = 0.28; *P* < 0.01) in the correlating matrix of all 22 immune cell proportions (Fig. 2) These results further indicate that anti-tumor immunity was activated with greater Tfh cell infiltration.

### 3.2 Association between the 22 types of TILs and clinical-pathological variables

**Figure 3:**
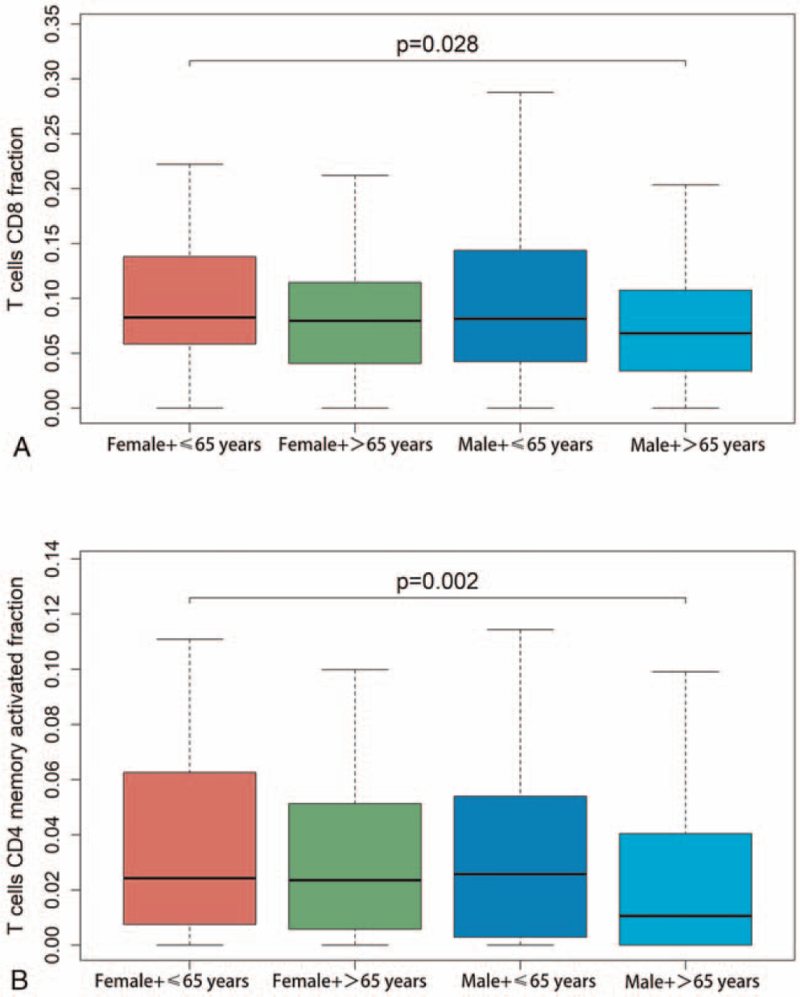
Association between 22 types of TILs and clinical-pathological variables. There was a higher proportion of CD8+ T cells (A) and memory activated CD4+ T cells (B) in younger women.

The correlation between the percentage of the 22 types of TILs with the clinical-pathological variables was further investigated. The percentage of CD4+ memory-activated T cells and CD8+ T cells were higher in females and patients aged ≤65 years (Fig. 3).

### 3.3 Prognosis prediction for the 22 types of TILs

**Figure 4:**
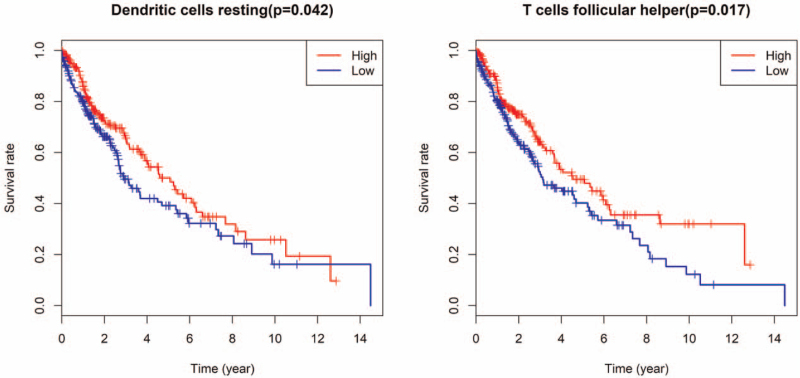
The overall survival associated with resting dendritic cells and follicular helper T cells. (A) The high percentage of Tfh cells were significantly associated with a favorable prognosis. (B) The high percentage of resting dendritic cells were also associated with a good prognosis, but this difference is barely noticeable.

We conducted a univariate Cox regression to analysis to screen for OS-related TILs. A K-M curve was subsequently drawn according to the percentage of each TILs. The high percentage of Tfh cells was significantly associated with a favorable prognosis. The high percentage of resting dendritic cells was also associated with a good prognosis, but this difference is barely noticeable (Fig. 4).

### 3.4 Construction of the ceRNA

**Figure 5:**
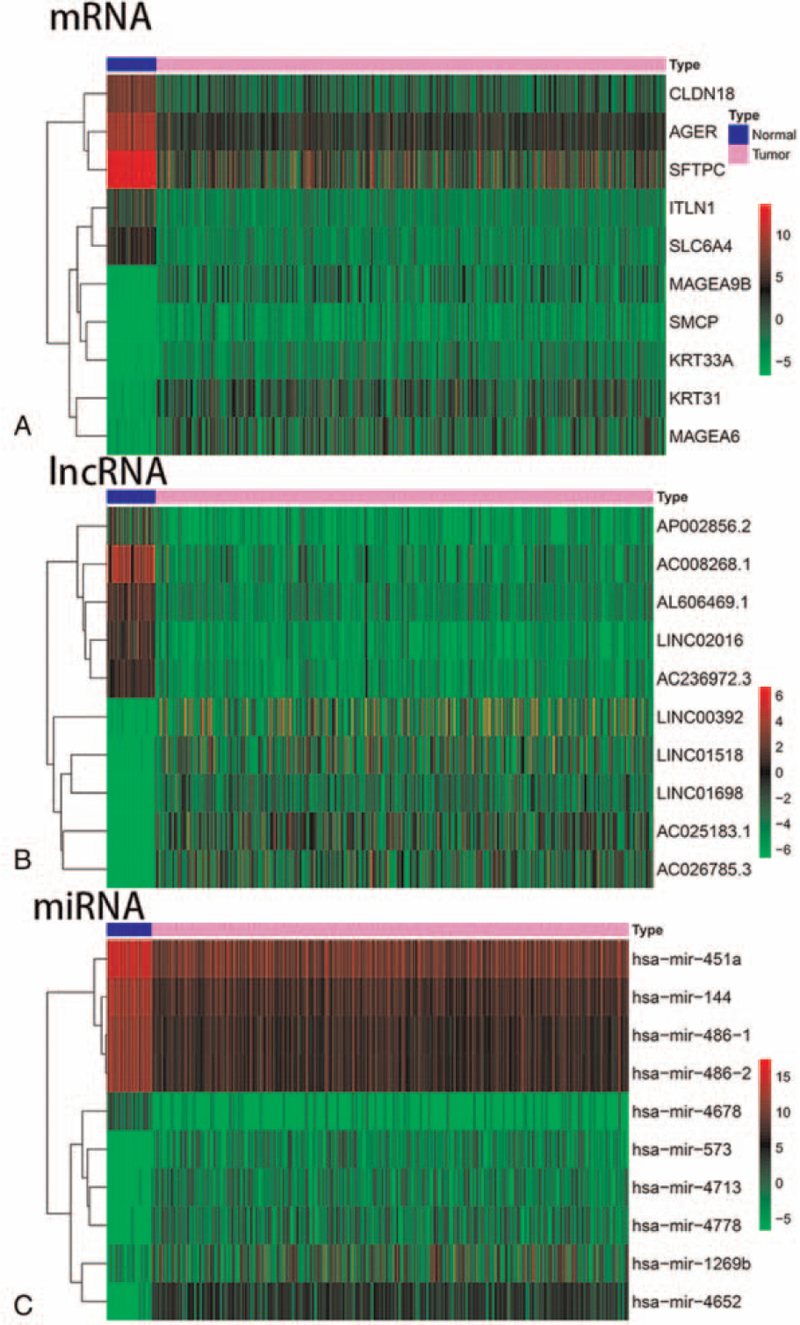
The heatmap to show the top 10 lncRNA, mRNA and miRNA with the most significant differences. (A) Top 10 differential mRNA. (B) Top 10 differential lnRNA. (C) Top 10 differential miRNA.

**Figure 6:**
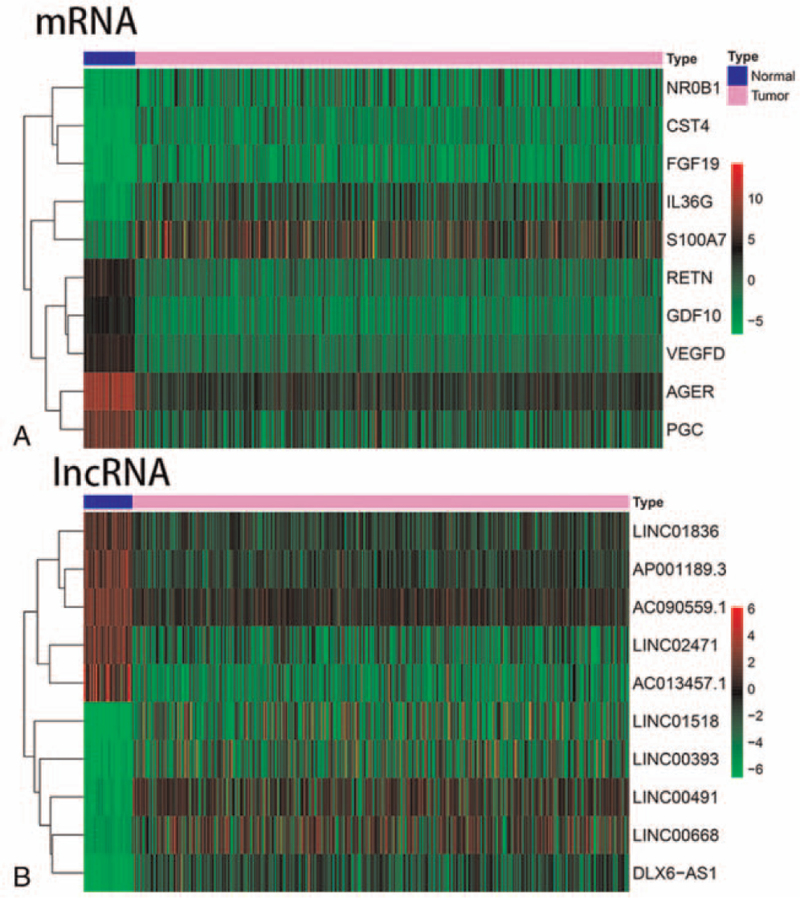
The top 10 immune related DEmRNA and DElncRNA were shown by heatmap. (A) Top 10 immune-related differential mRNA. (B) Top 10 immune-related differential lncRNA.

Using limma package, we obtained differentially expressed lncRNA, mRNA, and miRNA. We used heatmap to show the top 10 lncRNA, mRNA, and miRNA with the most significant differences(Fig. 5). Meanwhile, the top 10 immune-related DEmRNA and DElncRNA were also shown by heatmap (Fig. 6). As described in the methods, we created a ceRNA network composed of DEmRNA, DElncRNA, and DEmiRNA. As a result, 9 immune-related DEmRNA (TGFBR2, PROK2, NR4A2, FOS, GREM2 downregulated, ULBP2, FGFR3, and IL11 upregulated) were identified, 10 DEmiRNAs were upregulated and 19 DEmiRNA were downregulated. A total of 15 immune related DElncRNA (9 upregulated and 6 downregulated) were enrolled in the ceRNA network (Fig. 10). The results indicate that differentially expressed lncRNAs could indirectly interact with mRNAs though miRNA in SqCLC.

### 3.5 Prognosis prediction for DEmRNA, DElncRNA, and DEmiRNA in the ceRNA network

**Figure 7:**
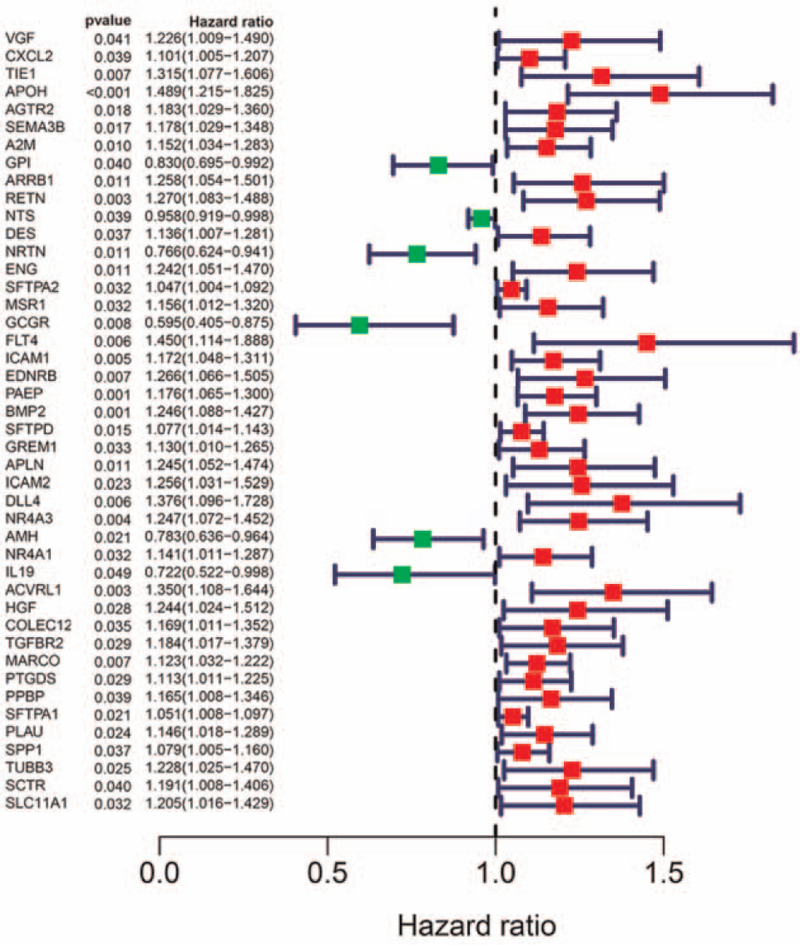
The univariate Cox regression analysis of immune-related mRNA. There were 44 immune-related mRNA significantly related to the overall survival.

**Figure 8:**
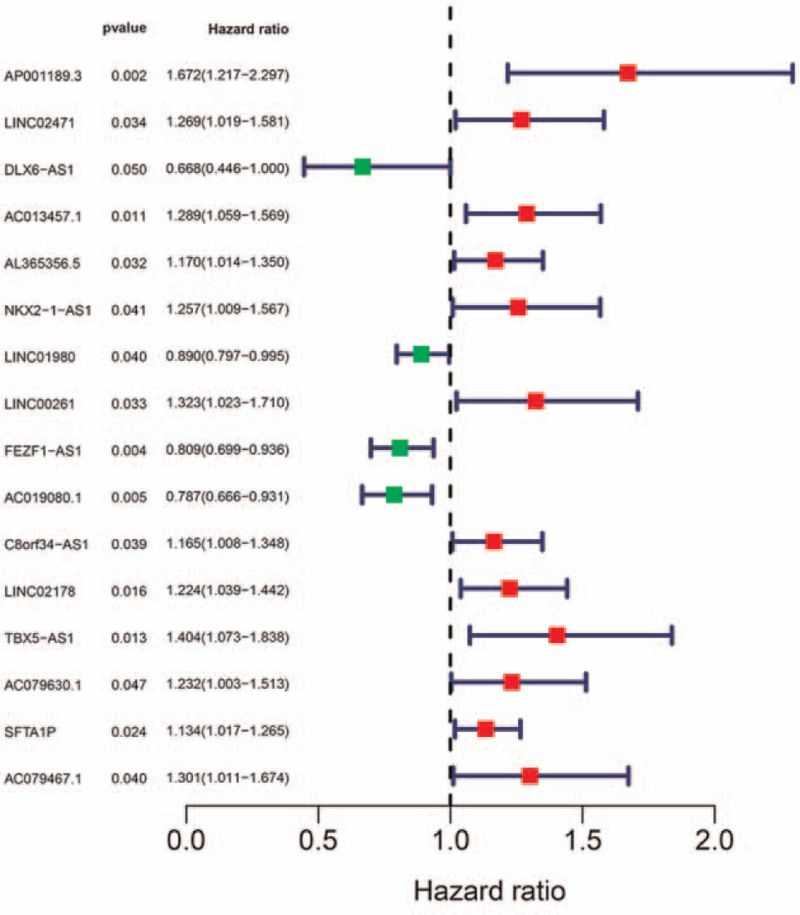
The univariate Cox regression analysis of immune-related lncRNA. There were 16 immune-related lncRNA significantly related to the overall survival.

**Figure 9:**
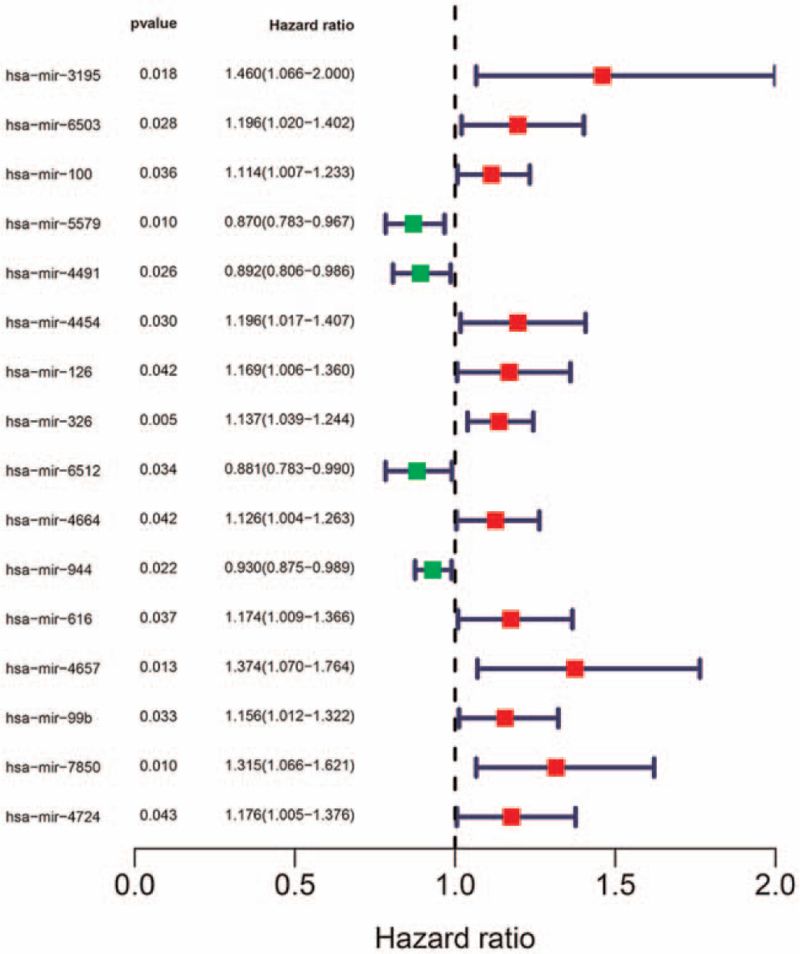
The univariate Cox regression analysis of miRNA. There were16 miRNA significantly related to the OS.

**Figure 10:**
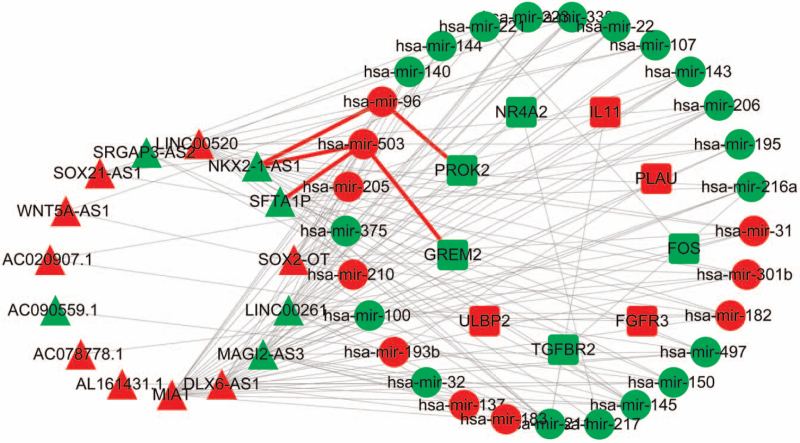
The ceRNA network in SqCLC. The down-regulated genes were presented in green, and the up-regulated genes were shown in red. The circle represented miRNA, the square represented mRNA, and the Triangle represented lncRNA. SFTA1P/NKX2-1-AS1, hsa-mir-503, GREM2 ceRNA axes and NKX2-1-AS1, hsa-mir-96, PROK2 ceRNA axe were linked by a red line.

A total of 495 SqCLCs have been used for mRNA and lncRNA sequencing, of which 469 SqCLCs were applied for miRNA sequencing. We conducted a univariate Cox regression analysis to screen for OS-related iDEmRNA, iDElncRNA, and DEmiRNA. Forty-four iDEmRNAs were significantly related to the OS (Fig. 7), and 16 iDElncRNAs were significantly related to the OS (Fig. 8). The upregulation of SFTA1P and NKX2-1-AS1 was related with poor prognosis in SqCLC compared with the down regulation of SFTA1P and NKX2-1-AS1 in SqCLC. There were 16 DEmiRNAs significantly related to the OS (Fig. 9). Further combination with the ceRNA network, DElncRNA (SFTA1P/NKX2-1-AS1), DEmiRNA (hsa-mir-503), DEmRNA (GREM2) ceRNA axes and DElncRNA (NKX2-1-AS1), DEmiRNA (hsa-mir-96), DEmRNA (PROK2) ceRNA axes were important for the immune status and prognosis of SqCLC (Fig. 10).

### 3.6 Association between TILs and ceRNA

**Figure 11:**
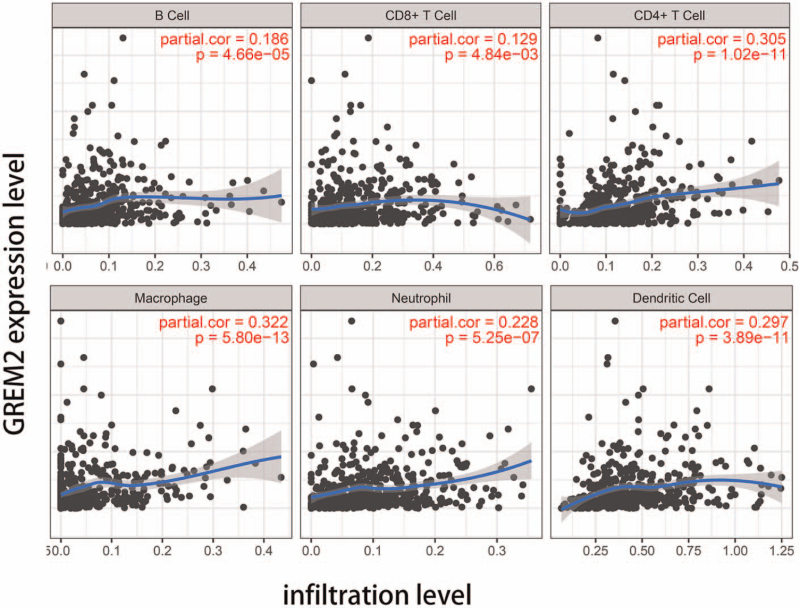
Association between TILs and GREM2. The expression level of GREM2 was significantly correlated with the infiltration levels of B cell, CD8+ T cell, CD4+ T cell, macrophage, neutrophil and dendritic cell, *P* < .05. TILSs = tumor infiltrating lymphocytes.

We analyze the relationship between GREM2 which was an important member in the ceRNA network and immune related cells. We found that the expression level of GREM2 significantly correlated with the infiltration level of B Cell, CD8+ T Cell, CD4+ T cell, macrophage, neutrophil and dendritic cell, as shown in Figure 11.

## 4 Discussion

In the present study, based on the deconvolutions of bulk gene expression data from the TCGA cohort, we found that there was a higher proportion of memory activated CD4+ T cells and CD8+ T cells in younger women. The high fraction of Tfh cells was significantly associated with a favorable prognosis. We identified the DElncRNA (SFTA1P/NKX2-1-AS1), DEmiRNA (hsa-mir-503), DEmRNA(GREM2) ceRNA axes and DElncRNA (NKX2-1-AS1), DEmiRNA (hsa-mir-96), DEmRNA (PROK2) ceRNA axes were associated with immune function and pathogenesis of SqCLC. In addition, SFTA1P/NKX2-1-AS1 in the ceRNA axes were significantly associated with patient OS.

Several studies have shown that CD4+ T cells can recognize tumor antigens presented on the surface of tumor cells and directly activate anti-tumor immunity associated with MHC class II molecules.^[9]^ Srivastava et al demonstrated that activated tumor-specific CD4+ T cells may be useful for the treatment of NSCLC.^[10]^ In addition, CD4+ T cells were found to correspond to sex and age in mice.^[11]^ Our findings revealed that there was a higher proportion of memory-activated CD4+ T cells in younger women. This finding may provide a suitable population for CD4 + T cell-related immunotherapy for SqCLC. DCs are antigen-presenting cells that function to process antigenic material and present it on the cell surface to T cells.^[12]^ Multiple studies have demonstrated that DCs used for immunotherapy can induce an immune response against NSCLC and prolong the OS time, without adverse effects.^[13,14]^ We found that resting DCs were associated with a better outcome. However, the prognosis of DCs in SqCLC requires further study.

Tfh cells may represent an important mechanism contributing to an exacerbated humoral response and autoantibody production.^[15]^ Tfh cells function as a critical regulator in several human malignancies. Infiltrating Tfh cells play a protective role in breast cancer.^[16]^ In ovarian cancer, Tfh cells are significantly enriched among TILs and are related to IL-21 and IL-10 secretion.^[17]^ Moreover, infiltrating Tfh cells participate in anti-tumor immunity in NSCLC patients, and are associated with favorable clinical outcomes.^[18,19]^ Our results were consistent with previous studies which show that Tfh cells were predictive of a good prognosis. NK cells can induce lung cancer regression, and NK cell immunotherapy has shown promising results.^[20]^ Our research has demonstrated that greater anti-tumor immunity was induced (including NK cells) as the number of infiltrating Tfh cells increased. Thus, Tfh cells may play an important role in killing squamous lung cancer cells by immune cells.

The dysregulation expression of lncRNA contribute to the biological functions of cancers, and lncRNA are becoming prognostic biomarkers in cancer.^[21]^ More and more evidences shown that lncRNA could bind with miRNAs, and act as endogenous miRNA sponges.^[22,23]^ In NSCLC, lncRNA KCNMB2-AS1 can sponge miRNA-374a-3p to facilitate the cancer progression.^[24]^ lncRNA LINC01246 could also sponge miRNA-519d-5p to promote NSCLC progression.^[25]^ Recent studies have demonstrated that lncRNAs play important roles in the development and differentiation of NSCLC.^[26]^ Dong and so on^[26]^ identified seven immune related lncRNA that could predict the prognosis of lung adenocarcinoma. However, only limited study has described the immune related lncRNA as ceRNA in SqCLC. In our study, we found that lncRNA (SFTA1P/NKX2-1-AS1), miRNA (hsa-mir-503), mRNA(GREM2) ceRNA axes and lncRNA (NKX2-1-AS1), miRNA (hsa-mir-96), mRNA (PROK2) ceRNA axes were associated with immune function and pathogenesis of SqCLC for the first time.

In addition, survival analysis indicated that lncRNA SFTA1P and NKX2-1-AS1 related with OS. Various studies have shown that SFTA1P is involved in the progression in many tumors.^[27–30]^ In papillary thyroid cancer (PTC), SFTA1P is the main constituents of competing endogenous lncRNA, and involved in the formation of PTC.^[21]^ Meanwhile, SFTA1P also action as competing endogenous RNA in head and neck squamous cell carcinoma.^[31]^ In hepatocellular carcinoma, AFTA1P could accelerate the tumor proliferation by down regulating mi4766-5p.^[32]^ Li et al^[33]^ and Xiong et al^[34]^ have shown that SFTA1P associated with prognosis and cisplatin chemosensitivity in lung squamous cell carcinoma. In gastric cancer, NKX2-1-AS1 is closely correlated with oncogenesis and progression.^[35]^ In human lung carcinoma, NKX2-1-AS1 could negatively regulated CD274/PD-L1, cell interaction genes, and cell migration.^[36]^ We reported that SFTA1P and NKX2-1-AS1 as an immune-related competing endogenous lncRNA in lung squamous cell carcinoma for the first time.

However, this article has some limitations. First, the clinical data were not perfect, no smoking history, brain metastasis, and liver metastasis data. Second, only lncRNAs in ceRNA network were significantly related with OS. Third, this study was based on the database and needs more basic experiments to further verify.

In summary, there were a higher proportion of CD8+ T cells and memory activated CD4+ T cells in younger women. Tfh cells were predictive of a good prognosis and reflected immune activation in SqCLC. SFTA1P/NKX2-1-AS1, hsa-mir-503,GREM2 ceRNA axes and NKX2-1-AS1, hsa-mir-96, PROK2 ceRNA axes were identified to be associated with the immune function, pathogenesis, and prognosis of SqCLC.

## Acknowledgments

The thank the authors who provided the TCGA and TIMER public data sets.

## Author contributions

Conceived and reviewed the literature: Zhihong Zhang and Hao Wang. Collected and analyzed the data: Zhihong Zhang, Hao Wang, Lijun Wang, Ke Xu, Yong Wang, Yehong Xu and Song Wei. Wrote the paper: Lijun Wang, Hao Wang. All authors read and approved the final manuscript.

**Conceptualization:** Hao Wang.

**Data curation:** Yehong Xu.

**Formal analysis:** Hao Wang.

**Resources:** Ke Xu, Yehong Xu, Song Wei, Yong Wang.

**Software:** Ke Xu, Yehong Xu, Song Wei, Yong Wang.

**Supervision:** MD, PHD Zhihong Zhang.

**Validation:** Song Wei, MD, PHD Zhihong Zhang.

**Visualization:** MD, PHD Zhihong Zhang.

**Writing – original draft:** Hao Wang, Lijun Wang.

**Writing – review & editing:** Hao Wang, Lijun Wang.

## References

[1] Hirsch FR Kerr KM Bunn PA Jr. Molecular and immune biomarker testing in squamous-cell lung cancer: effect of current and future therapies and technologies. Clin Lung Cancer 2018;19:331–9. 29773328 10.1016/j.cllc.2018.03.014

[2] Yu H Chen Z Ballman KV. Correlation of PD-L1 expression with tumor mutation burden and gene signatures for prognosis in early-stage squamous cell lung carcinoma. J Thorac Oncol 2019;14:25–36. 30253973 10.1016/j.jtho.2018.09.006 PMC6309634

[3] Ku BM Kim Y Lee KY. Tumor infiltrated immune cell types support distinct immune checkpoint inhibitor outcomes in patients with advanced non-small cell lung cancer. Eur J Immunol 2021;51:956–64. 33506525 10.1002/eji.202048966 PMC8248238

[4] Parra ER Behrens C Rodriguez-Canales J. Image analysis-based assessment of PD-L1 and tumor-associated immune cells density supports distinct intratumoral microenvironment groups in non-small cell lung carcinoma patients. Clin Cancer Res 2016;22:6278–89. 27252415 10.1158/1078-0432.CCR-15-2443 PMC5558040

[5] Ho KH Chang CJ Huang TW. Gene landscape and correlation between B-cell infiltration and programmed death ligand 1 expression in lung adenocarcinoma patients from The Cancer Genome Atlas data set. PLoS One 2018;13:e0208459. 30521597 10.1371/journal.pone.0208459 PMC6283571

[6] Tsakonas G Lewensohn R Botling J. An immune gene expression signature distinguishes central nervous system metastases from primary tumours in non-small-cell lung cancer. Eur J Cancer 2020;132:24–34. 32325417 10.1016/j.ejca.2020.03.014

[7] Hwang S Kwon AY Jeong JY. Immune gene signatures for predicting durable clinical benefit of anti-PD-1 immunotherapy in patients with non-small cell lung cancer. Sci Rep 2020;10:643. 31959763 10.1038/s41598-019-57218-9 PMC6971301

[8] Chen B Khodadoust MS Liu CL Newman AM Alizadeh AA. Profiling tumor infiltrating immune cells with CIBERSORT. Methods Mol Biol 2018;1711:243–59. 29344893 10.1007/978-1-4939-7493-1_12 PMC5895181

[9] Haabeth OA Tveita AA Fauskanger M. How do CD4(+) T cells detect and eliminate tumor cells that either lack or express MHC class II molecules? Front Immunol 2014;5:174. 24782871 10.3389/fimmu.2014.00174 PMC3995058

[10] Srivastava MK Bosch JJ Thompson JA Ksander BR Edelman MJ Ostrand-Rosenberg S. Lung cancer patients’ CD4(+) T cells are activated in vitro by MHC II cell-based vaccines despite the presence of myeloid-derived suppressor cells. Cancer Immunol Immunother 2008;57:1493–504. 18322683 10.1007/s00262-008-0490-9 PMC2805175

[11] Arsenovic-Ranin N Kosec D Pilipovic I. Sex and age as determinants of rat T-cell phenotypic characteristics: influence of peripubertal gonadectomy. Mol Cell Biochem 2017;431:169–85. 28281185 10.1007/s11010-017-2989-x

[12] Wang X Tang S Cui X. Cytokine-induced killer cell/dendritic cell-cytokine-induced killer cell immunotherapy for the postoperative treatment of gastric cancer: a systematic review and meta-analysis. Medicine 2018;97:e12230. 30200148 10.1097/MD.0000000000012230 PMC6133452

[13] Zhang L Yang X Sun Z. Dendritic cell vaccine and cytokine-induced killer cell therapy for the treatment of advanced non-small cell lung cancer. Oncol Letters 2016;11:2605–10. 10.3892/ol.2016.4273 PMC4812113 27073525

[14] Zhao P Bu X Wei X. Dendritic cell immunotherapy combined with cytokine-induced killer cells promotes skewing toward Th2 cytokine profile in patients with metastatic non-small cell lung cancer. Int Immunopharmacol 2015;25:450–6. 25698555 10.1016/j.intimp.2015.02.010

[15] Mesquita D Jr Cruvinel WM Resende LS. Follicular helper T cell in immunity and autoimmunity. Braz J Med Biol Res 2016;49:e5209. 27096200 10.1590/1414-431X20165209 PMC4843212

[16] Gu-Trantien C Loi S Garaud S. CD4(+) follicular helper T cell infiltration predicts breast cancer survival. J Clin Invest 2013;123:2873–92. 23778140 10.1172/JCI67428 PMC3696556

[17] Li L Ma Y Xu Y Maerkeya K. TIM-3 expression identifies a distinctive PD-1(+) follicular helper T cell subset, with reduced interleukin 21 production and B cell help function in ovarian cancer patients. Int Immunopharmacol 2018;57:139–46. 29482158 10.1016/j.intimp.2018.02.016

[18] Liu X Wu S Yang Y Zhao M Zhu G Hou Z. The prognostic landscape of tumor-infiltrating immune cell and immunomodulators in lung cancer. Biomed Pharmacother 2017;95:55–61. 28826097 10.1016/j.biopha.2017.08.003

[19] Ma QY Huang DY Zhang HJ Chen J Miller W Chen XF. Function of follicular helper T cell is impaired and correlates with survival time in non-small cell lung cancer. Int Immunopharmacol 2016;41:01–7. 10.1016/j.intimp.2016.10.014 27788370

[20] Aktas ON Ozturk AB Erman B Erus S Tanju S Dilege S. Role of natural killer cells in lung cancer. J Cancer Res Clin Oncol 2018;144:997–1003. 29616326 10.1007/s00432-018-2635-3 PMC11813529

[21] Jiang Y Wang J Chen J Wang J Xu J. Construction and analysis of an aberrant lncRNA-miRNA-mRNA network associated with papillary thyroid cancer. Medicine 2020;99:e22705. 33157921 10.1097/MD.0000000000022705 PMC7647549

[22] Li F Huang C Li Q Wu X. Construction and comprehensive analysis for dysregulated long non-coding RNA (lncRNA)-associated competing endogenous RNA (ceRNA) network in gastric cancer. Med Sci Monit 2018;24:37–49. 29295970 10.12659/MSM.905410 PMC5761711

[23] Zhao Y Wang H Wu C. Construction and investigation of lncRNA-associated ceRNA regulatory network in papillary thyroid cancer. Oncol Rep 2018;39:1197–206. 29328463 10.3892/or.2018.6207 PMC5802034

[24] Yang H Wang Z Wang Z. Long noncoding RNA KCNMB2-AS1 increases ROCK1 expression by sponging microRNA-374a-3p to facilitate the progression of non-small-cell lung cancer. Cancer Manag Res 2020;12:12679–95. 33335424 10.2147/CMAR.S270646 PMC7737946

[25] Dai J Wang B Zhao Y. Long noncoding RNA LINC01426 sequesters microRNA-519d-5p to promote non-small cell lung cancer progression by increasing ETS1 expression. Cancer Manag Res 2020;12:12697–708. 33335425 10.2147/CMAR.S277113 PMC7736839

[26] Sun J Zhang Z Bao S. Identification of tumor immune infiltration-associated lncRNAs for improving prognosis and immunotherapy response of patients with non-small cell lung cancer. J Immunother Cancer 2020;8:10.1136/jitc-2019-000110 PMC7057423 32041817

[27] Huang GQ Ke ZP Hu HB Gu B. Co-expression network analysis of long noncoding RNAs (IncRNAs) and cancer genes revealsSFTA1P and CASC2abnormalities in lung squamous cell carcinoma. Cancer Biol Ther 2017;18:115–22. 28118064 10.1080/15384047.2017.1281494 PMC5362987

[28] Li S Chen X Liu X. Complex integrated analysis of lncRNAs-miRNAs-mRNAs in oral squamous cell carcinoma. Oral Oncol 2017;73:01–9. 10.1016/j.oraloncology.2017.07.026 28939059

[29] Zhao J Xu J Shang AQ Zhang R. A Six-LncRNA Expression Signature Associated with Prognosis of Colorectal Cancer Patients. Cell Physiol Biochem 2018;50:1882–90. 30396175 10.1159/000494868

[30] Liao HT Huang JW Lan T. Identification of the aberrantly expressed LncRNAs in hepatocellular carcinoma: a bioinformatics analysis based on RNA-sequencing. Sci Rep 2018;8:5395. 29599483 10.1038/s41598-018-23647-1 PMC5876391

[31] Zhang C Cao W Wang J. A prognostic long non-coding RNA-associated competing endogenous RNA network in head and neck squamous cell carcinoma. PeerJ 2020;8:e9701. 32983633 10.7717/peerj.9701 PMC7500352

[32] Huang G Yang Y Lv M. Novel lncRNA SFTA1P promotes tumor growth by down-regulating miR-4766-5p via PI3K/AKT/mTOR signaling pathway in hepatocellular carcinoma. Onco Targets Ther 2020;13:9759–70. 33061455 10.2147/OTT.S248660 PMC7533222

[33] Li L Yin JY He FZ. Long noncoding RNA SFTA1P promoted apoptosis and increased cisplatin chemosensitivity via regulating the hnRNP-U-GADD45A axis in lung squamous cell carcinoma. Oncotarget 2017;8:97476–89. 29228625 10.18632/oncotarget.22138 PMC5722577

[34] Xiong Y Zhang X Lin Z. SFTA1P, LINC00968, GATA6-AS1, TBX5-AS1, and FEZF1-AS1 are crucial long non-coding RNAs associated with the prognosis of lung squamous cell carcinoma. Oncol Lett 2019;18:3985–93. 31579094 10.3892/ol.2019.10744 PMC6757264

[35] Wang J Ding Y Wu Y Wang X. Identification of the complex regulatory relationships related to gastric cancer from lncRNA-miRNA-mRNA network. J Cell Biochem 2020;121:876–87. 31452262 10.1002/jcb.29332

[36] Kathuria H Millien G McNally L. NKX2-1-AS1 negatively regulates CD274/PD-L1, cell-cell interaction genes, and limits human lung carcinoma cell migration. Sci Rep 2018;8:14418. 30258080 10.1038/s41598-018-32793-5 PMC6158174

